# Embodied experiences of lived citizenship beyond the European privilege. The case-study of Romanian women farmworkers in eastern Sicily

**DOI:** 10.3389/fsoc.2025.1558541

**Published:** 2025-08-08

**Authors:** Monica Massari, Gianluca Gatta, Simona Miceli, Federica Cabras

**Affiliations:** Department of International, Legal, Historical and Political Studies, University of Milan, Milan, Italy

**Keywords:** lived citizenship, migration, gender, Europe, exploitation, agriculture, agency, isolation

## Abstract

**Introduction:**

The article examines the conditions of life and agency of Romanian migrant farmworkers in Sicily's agricultural sector, focusing on the migration-citizenship nexus through a gendered, embodied, racialized, and intersectional lens. It aims to shed light on the paradoxes and contradictions still affecting both formal and lived forms of European citizenship regimes.

**Method:**

This qualitative study is based on fieldwork carried out between 2022 and 2024, which included informal meetings, interviews with migrant women, employers, trade unionists, health and social workers, and participant observation in an after-school center for migrant children.

**Results:**

The results show that Romanian women face multiple vulnerabilities due to poor working and living conditions, physical and social isolation, gender, family situation, and legal status. Their European citizenship paradoxically subjects them to worse conditions than other migrant workers. Despite their precarious situations, these women display agency practices and counteractions in their daily lives, mainly through micro-strategies that might at first glance appear discreet and passive yet, at the same time, are the outcome of their conscious capacity to adapt to potentially unbearable circumstances. They also employ reworking strategies, such as quitting jobs, and occasionally engage in long-term acts of rupture to claim rights and challenge power structures.

**Discussion:**

The analysis contributes to the debate on how structural determinants of oppression and exploitation relate to subjectivity and agency. It explores various forms of counteraction and survival through the concept of lived citizenship, novel ways of experiencing and enacting citizenship within and across different contexts and spaces, both physical and symbolic. While these actions are neither striking nor highly organized or effective in achieving emancipation, they serve as responses and countermeasures within a restrictive system and shed light on migrant women's reflexivity and struggle for autonomy and control over their lives that deserve greater attention.

## 1 Introduction

In the context of international migration, citizenship often stands as one of the last borders that people cross, and sometimes miss, in their attempt to settle in destination societies. However, it is not exclusively a status to be acquired, since citizenship unfolds along a plurality of dimensions, i.e., formal, lived, affective and enacted. Indeed, critically addressing it from the perspective of migration allows us to overcome the narrow dichotomy between citizens and foreigners and shedding light on the continuum along which ambivalent and simultaneous experiences of inclusion and exclusion take shape. We argue that adopting a gendered, embodied, racialized and intersectional approach to exploring the nexus between migration and citizenship enables us to highlight the paradoxes that still affect citizenship within situated empirical contexts. As research on lived citizenship has extensively emphasized (Lister, 2007; McNevin, 2011; Kallio et al., 2020; Fjetland et al., 2022), focusing only on the analysis of the formal dimension of citizenship prevents us from grasping how people who are excluded experience, contest and claim it, but also how it may be undermined and not actually enjoyed by those who, at least in part, hold it. While lived citizenship has been widely explored—particularly in the wake of feminist theories (Yuval-Davis, 1997; Lister, 2003; Lister et al., 2007; Siim and Stoltz, 2024) and, more recently, within the interpretative framework on acts of citizenship (Isin, 2008)—the paradoxes experienced by formal citizens (Gatti, 2022) remain more elusive. This is partly because the possession of formal citizenship can overshadow experiences of discrimination and marginalization, while citizenship studies have often focused on urban contexts as primary sites of political struggle and claims-making (Müller, 2022a). As a result, nonurban settings—where physical and social isolation frequently intersect with multiple forms of marginalization—have been comparatively overlooked. Hence, this study's focus on a rural context provides an original lens through which to examine citizenship paradoxes, setting it apart from earlier contributions in the field.

In this article, we address the case study of Romanian migrant women farmworkers in the rural context of Southern Italy. While these women are formally recognized as part of a common European citizenship, they experience severe forms of labor and sexual exploitation, navigating conditions of extreme economic and social marginality and isolation. The case of European citizenship is in fact quite complex, since taking for granted the equivalence between European and EU citizenship favored a focus on legal status (Isin and Saward, 2013), emphasizing how Eastern EU enlargement processes effectively expanded the number of people who can move freely within its borders. However, EU citizenship is a form of derivative citizenship that is added to the national one, keeping intact the differences between “multiple Europes,” i.e., between those who are actually considered European and those who are not (Boatcă, 2010).

Thus, embracing a questioning stance on European citizenship, our analysis aims to show the extent to which the European citizenship held by these women is only one piece in the complex puzzle in which power relations influenced by gender, class and otherness dynamics intervene and where various forms of exploitation and micro-practices of survival may coexist.

The structure of the article is as follows. In the next section we outline our theoretical background, which draws both on the critical debate on formal, legal and lived citizenship, with particular attention to its transnational and spatial dimension, the relationship between citizenship and gender and the *othering* of Eastern Europe, with a focus on racialization and sexualization processes addressing white migrants, especially women. Then we describe our empirical context and methodological framework. In the following section, we present our findings that focus on the living and working conditions of Romanian farmworkers in greenhouses in Sicily and their agency practices which revolve around three main strategies, i.e., reflexive flexibility, escape and rupture. A discussion and conclusive section close the article.

## 2 Theoretical background

A “mutually constitutive relationship” (Džankić and Vink, 2022, p. 359) links migration and citizenship, as the status of being a citizen heavily influences migratory opportunities and serves as an object of aspiration, while the discourse on migration shapes citizenship policies. However, the conception of citizenship confined to its formal dimension as a legal status—comprising a set of attributes that regulate rights and responsibilities within a given political space—fails to fully capture how citizenship is experienced, contested, and claimed in the informal interstices of the social fabric, particularly by those excluded from formal political participation. In response to this need, since the mid-1990s, a body of research has developed around the concept of citizenship, aiming to rework it, extend its inclusive potential, and provide new emancipatory tools for social groups traditionally excluded from the normative notion of citizenship (Lister, 2007). Beyond nation-state and status-centered theories of citizenship, new approaches to citizenship as a social process have emerged, focusing on “substantive citizenship” and contributing to a “(…) sociologically informed definition of citizenship in which the emphasis is less on legal rules and more on norms, practices, meanings, and identities” (Isin and Turner, 2002, p. 4). The perspectives of marginalized sectors of society and the meanings of citizenship emerging “from below” have been discussed (Kabeer, 2005). A more inclusive and expansive understanding of citizenship has been claimed, requiring a less abstract approach to the notion and consideration of the situated nature of its expression and its rootedness in actual socio-political spaces, where different scales intersect below and above the nation-state. Consequently, against the abstraction of normative approaches to citizenship, both the exploration of “new geographies of citizenship” (Desforges et al., 2005; Staeheli, 2011; Stokke and Erdal, 2017), which focuses on the performance of citizenship across multiple spatial scales from the intimate to the global, and the feminist attentiveness to embodied forms of citizenship, have contributed to the emergence of “a more grounded understanding of citizenship as a practice” (Lister, 2007, p. 55).

### 2.1 Transnational lived citizenship and socio-spatial isolation

The peculiar position of the population at the center of our analysis, which cuts across the divide between formal and informal, included and excluded, internal and external, in a highly oppressive context that is also spatially distant from the stages where political life is visible, prompted us to question the meaning of a series of practices, stances, acts, and discourses that appear as disturbances to the clear-cut image of vulnerable people as passive objects of oppression. Aware that these experiences and forms of agency are not directly transformative, we nevertheless wondered if they were merely fables and desperate conditioned responses within highly uneven power relations, or if they might somehow question and contribute to reframing the migration-citizenship nexus.

To address these questions, we draw on the concept of “lived citizenship” and its developments (Kallio et al., 2020; Lister, 2003, 2007; Lister et al., 2007). This framework, with its focus on people's standpoints and citizenship practices, helps us grasp the political meaning of our data. Aiming to explore and make visible the implications of being and feeling like a citizen in people's daily lives within specific contexts, and drawing on everyday life studies, this approach emphasizes contexts where citizenship is experienced and understood by individuals in their daily lives, thus not limited to fixed statuses or state boundaries. This perspective allows us to understand how our research participants make meaning of citizenship within the concrete social, cultural, and material constraints that shape their lives. It also helps us see how they understand and negotiate the power relations they experience and the rights to which they are entitled.

The application of a lived citizenship perspective to migration—since its spread from childhood and poverty studies to other social domains—proved to be very effective, since migration is an area where the gap between formal and informal types of political participation is particularly evident (McNevin, 2006, 2011). Several aspects of the relationship between migration and lived citizenship have been studied, i.e., migration and gender (Cherubini, 2011); asylum seeking and irregular migration (Cochrane and Wolff, 2022); the relationship of transnational lived citizenship with diaspora (Müller, 2023; Müller and Belloni, 2021; Pascucci, 2016), second generations (Calabretta, 2023), and activism (Müller, 2022a); cross-border migration and political subjectivity (Palma-Gutiérrez, 2023); refugee children and deportability (Fichtner and Trân, 2020); labor market integration (Müller, 2022b); and EU and non-EU skilled migration (Sönmez Gioftsios and Özçürümez, 2024).

And even for our understanding of our research participants' experiences as European citizens and foreign workers, the redefinition of the concept of political participation that this framework has enacted is crucial. It implies a vision of citizenship that transcends issues of status and engages with positions and identities, emphasizing agency expressed through seemingly small political acts in domestic and informal settings by individuals perceived as vulnerable and who traditionally engage in citizenship outside of formal political arenas (Fjetland et al., 2022; Lister et al., 2003; Lister, 2007).

Moreover, as the genealogy of “lived citizenship” in feminist research led to challenging the gender bias inherent in normative approaches to citizenship, which are anchored in the link between citizenship and the public sphere, and the public-private dichotomy (Lister, 2003; Voet, 1998; Vogel, 1991; Walby, 1994), and opened up space for recognizing private, domestic, and intimate spheres as political (Plummer, 2003), this perspective allows us to conceptualize the gendered dimension of the phenomena we have observed.

Lived citizenship is a multidimensional concept (Kallio et al., 2020). It is inherently tied to the spatial and temporal contexts in which it is experienced. However, due to global transformations, citizenship has also taken on new forms at various scales, both local and transnational, which must be carefully considered when studying transnational migration, as in our case. Indeed, transnational mobility has intensified the formation of bonds and identifications at different scales, where diversifying forms of participation are closely linked to interpersonal and emotional relationships. These processes shape citizenship in specific spatial locations and within a set of relationships—interpersonal and intergenerational—that define belonging and participation, both through formal political channels and in everyday social life, where public/institutional and private/individual spheres intersect. Moreover, citizenship can be constructed, claimed, and materialized through performative practices at various scales and in different places, beyond just being a status.

Therefore, the concept is broad enough to allow us to connect in our analysis a range of acts and reactions that span from more direct forms of agonistic resistance to forms of mutual and self-care, based on intersubjectivity and affectivity. Indeed, the affective dimension of agency is often undervalued in the status approach to citizenship, while the “lived” approach emphasizes the strong connection between the feeling of belonging and the emotions tied to being and feeling like a citizen, especially within the neoliberal state where the concept of “care” plays a central role. This approach allows the lived experience of citizenship to be examined in less conventional contexts and forms, while establishing criteria to distinguish political actions—aimed at shaping society around significant issues, often outside formal political participation channels—from non-political ones.

For the sake of our discourse, particular attention is required to the spatial dimension of lived citizenship, due to the extreme social and spatial isolation experienced by our research participants as marginalized transnational European citizens. In this regard, we find the concept of “transnational lived citizenship” useful. This concept combines the idea of lived citizenship with the transnational approach to migration studies (Kallio and Mitchell, 2016; Martin and Paasi, 2016) and challenges state language on migration—the distinction between migrants and refugees, or the various categories of migrants—by focusing on the intersections between several geographic scales and types of mobility. Concentrating on the figure of the migrant as pivotal in contemporary world dynamics, and intertwining legal definitions, belonging, aspiration and activism in dealing with and going beyond the nation-state, this strand of research has been particularly attentive to the urban dimension of transnational lived citizenship, which is conceived in terms of *city-zenship*, with the city being the quintessential place where struggles for rights and political solidarity emerge (Müller, 2022a).

In our case study, however, we would like to shift our attention to the relationship between transnational lived citizenship and socio-spatial isolation, since our research expressly focused on migrant farmworkers working and living in remote and secluded rural areas, a condition that increases their marginalization. With the overarching centrality that mobility and interconnectivity have gained in contemporary social theory, both socio-spatial isolation (Atkinson, 2009), and immobility have been underestimated (Salazar, 2021; Schewel, 2020). However, recent developments have acknowledged that, often, immobility is part of the experience of mobility, which may include being stuck and constrained (Bélanger and Silvey, 2020). Hence, the mobility-immobility dynamic, intertwined with economic rationalities, racialization practices and gendered vulnerabilities, is central to understanding the differentiated experiences of exploitation, agency, and lived citizenship. In our contribution we would like to combine (im)mobility and socio-spatial isolation—an expression we prefer in order to avoid the city-centric perspective of the literature on social and spatial “segregation” (Pereira, 2021; Piekut, 2021)—to see how transnational mobility and immobility can occur between isolated spaces, without involving forms of active, albeit informal participation, but nevertheless presenting elements of everyday survival and resistance which can be fruitfully understood through the lenses of lived citizenship.

### 2.2 Gender, citizenship and the Eastern European other

The relationship between legal and lived citizenship is not static and takes on different configurations depending on the actual social contexts, related social divisions, the subjects involved, the constraints they face, and the agency practices they exercise. This is particularly relevant in the field of migration research, where growing attention goes to all situations where citizenship is enacted and lived despite its formal absence (Gatti, 2023). Moreover, due to the seminal contribution of the feminist framework in critically exploring these situated dynamics between legal and lived citizenship, the central role of gender in structuring different access to citizenship rights and experiences of citizenship has been extensively analyzed since the 1990s (Lister, 1997; Yuval-Davis, 1997). This analysis deconstructs the idea that citizenship is a generator of equalities and rights and emphasizes that its supposed universalism is not only jeopardized by class inequalities (Marshall, 1950), but also by gender differences that strongly affect the enjoyment of a full citizenship, although migrant women tirelessly try to cross its boundaries (Tastsoglou and Dobrowolsky, 2006; Yuval-Davis, 2007; Erel, 2009).

A particularly intricate case involves intra-European migration along the East/West axis. The EU's processes of eastward enlargement in 2004 and 2007 considerably extended the pool of European citizens, granting freedom of movement to people who previously had to use international migration channels. However, in several cases, the recognition of certain rights linked to European citizenship (formal dimension) is combined with the deployment of new forms of exploitation for those who continue to be perceived as migrants (lived experience). In this regard, feminist research has been crucial in unpacking and bringing into focus some “neglected sides of European citizenship” (Aksola, 2012, p. 342). It has emphasized, for instance, how global care chains (Andall, 2003; Ehrenreich and Hochschild, 2003) are sustained within the EU through a system of differentiated lived citizenship. This system contrasts Western European women—who have increasingly entered the labor market and the public sphere—with Eastern European women, who have migrated westward to take on the burden of domestic and care work (Aksola, 2012). In doing so, they not only support Western households but also ensure the survival of their families and countries of origin (Sassen, 2002).

Just to give an example, if affluent men can exchange global mobility for a paycheck or an investment, poor women and racialized people can only exchange their bodies, literally offering themselves and their emotional and caring labor in return for mobility through citizenship (Boatcă and Roth, 2016, p. 17). In the case of Romanian migrant women farmworkers addressed in this article, their status as European citizens—as our analysis emphasizes—does not always protect them from multiple forms of exploitation, so they actually experience a sort of “lessened citizenship” (Karagiannopoulou et al., 2024). Hence, to unpack European citizenship in the lived experience of these women, we consider it essential to refer to the transnational and intersectional turn in gender and citizenship research, a further step for theorizing differences and inequalities, moving citizenship and gender research beyond the focus on nation-state and gender boundaries (Siim and Stoltz, 2024). Moreover, to avoid the risk of endorsing a Eurocentric analysis of gender and citizenship, as postcolonial and black feminist thought has extensively and long since emphasized (Mohanty, 1984; Anzaldua, 1987; Hooks, 1990), it is necessary to grasp how gender differences intersect with other inequalities in concrete social contexts, so as to rethink the condition of citizen in light of these exclusionary intersections (Yuval-Davis, 2015) and not only as a formal status. If intersectionality is always situated (Yuval-Davis, 2011; Hancock, 2016; Hill Collins, 2019), so too is citizenship, especially in its lived dimension. Indeed, widening the gap between formal and lived citizenship, various factors intervene that position people within social contexts along hierarchical scales that do not entirely correspond to the legal status and rights those same people enjoy.

Within migration processes, a particularly relevant variable that intersects with gender is otherness, which plays a crucial role in the stigmatizing and exclusionary processes affecting migrants in Europe. Otherness can be associated with visible markers (Guillaumin, 1992), such as certain somatic traits, skin color or religious affiliation, thus producing blatant racialization processes, especially in the case of migration from the global south (Erel et al., 2016). Alternatively, it may derive from markers that are more difficult to detect since they occur in the sphere of so-called whiteness, such as nationality in the case of intra-European migration or belonging to specific ethnic minorities historically discriminated in Europe (i.e., Roma people), engendering quite ambiguous phenomena of racialization. Whiteness is a social construction that results in a structural position of advantages and privileges generally experienced by white people (Frankenberg, 1993), which, however, (white) migrants from Eastern Europe do not always enjoy (Krivonos, 2018). Indeed, within the destination country, they are often perceived as “other whites,” meaning that while they enjoy the racial privilege of being able to pass partially unnoticed as white, they suffer the national disadvantage of being from a non-Western country (Blachnicka-Ciacek and Budginaite-Mackine, 2022). While Eastern Europeans with strong cultural and economic capital can negotiate their position and obtain certain privileges, for the working class, international migration implies “the risk of downward racial mobility through reclassification as non-white” (Boatcă, 2017, p. 470). These processes need to be understood in the light of a cognitive map with much older historical roots, which has sustained a kind of “moral geography” of the continent: a scale ranging from the western parts of Europe, modern and democratic, to an area labeled as violent, backward and inferior, usually located in the Balkans, whose Europeanness is questioned (Boatcă, 2010). The outcome is the construction of Eastern Europe as an “internal other” (Todorova, 1997). Although the criterion of “race,” which was central to colonial classifications, was not explicitly used for the construction of the differences between the two parts of Europe, the discursive strategies deployed functioned mainly through the use of dichotomies, such as “civilization/barbarism,” “rational/irrational,” and the idea that there are “pathological regions” within the “North” and the “West” (Boatcă, 2007).

The condition of *other* whiteness thus is also shaped by socio-cultural and religious differences that, when interpreted through the lens of ethnicity, produce internal distinctions within the broader category of Eastern Europeans, often relegating certain groups to the bottom of both national and transnational hierarchies. Especially in contexts of migration, where boundaries of belonging are negotiated as well as reinforced, ethnicity is not a fixed attribute, but a socially constructed and relational category, produced through everyday interactions, labor dynamics, and gender construction (Anthias, 2002). Concerning our research case, the ethnic identity of Romanian migrant women is often ambiguous and selectively silenced; in particular, belonging to the Roma minority—highly stigmatized both in Romania and in Italy—is rarely made explicit.

Consequently, researching intra-European migration requires considering the extent to which these epistemological processes affect the living conditions of many Eastern European migrants in Western countries (Lundström, 2017). In this framework, the eastward enlargement of the European Union has produced further stratification in the migrant labor force, as citizens from Eastern European countries have been subjected to processes of stigmatization that have weakened both their whiteness-related advantages and the freedom of movement guaranteed by European citizenship (Caruso, 2016; Grappi and Sacchetto, 2013).

Romanians, who have been the largest foreign group in Italy since the early 2000s (Centro Studi e Ricerche IDOS, 2022), have especially been the target of several stereotyping and stigmatization campaigns by the Italian media, particularly in connection with Romania's entry into the EU in 2007. As Cingolani (2009) points out, within Italian society it is possible to trace two opposing social representations, both gendered. The first, of an assimilationist kind, presents Romanians as the “more integrable” immigrants as they are white and Christian, where men are tireless workers and women maintain the femininity and respect for male authority that Italian women have lost (Cingolani, 2009, p. 55). On the other hand, a criminalizing representation has been reinforced, supported by a strong anti-Romanian sentiment, which depicts men as violent, dedicated to illegal activities such as theft and the exploitation of sex work, and women as skilled at sexually *corrupting* Italian men, willing to do anything to obtain advantages (Cingolani, 2009, p. 56). These deteriorated media and common-sense representations, with overlapping processes of sexualization and racialization, apply not only to Romanian women but more generally to “women from the East,” often represented as “overly sexualised, traditional and eroticised subjects,” with the aim of emphasizing the presumed gender equality achieved in Western Europe (Krivonos and Diatlova, 2020, p. 120). This specific imaginary—shaped by gender stereotypes and the construction of a suspicious otherness—underpins a subtle yet persistent stigmatization of Romanian women. It exemplifies a form of exclusionary intersectionality that affects the lives of Romanian women working in the Sicilian countryside, potentially undermining their status as European citizens, both in terms of identity and rights. Indeed, although the migration journey of those who have the right to move and reside in a country is different from those who do not, it is not without costs. Citizenship status interacts with other factors, with the consequence that “EU citizenship is part of an international division of labor, implying gender, race, class and nationality and entailing increasing inequalities between groups of women (and also men)” (Aksola, 2012, p. 353). Thus, although Romanian women farmworkers hold European citizenship, their simultaneous positioning as women, as Romanian nationals, and as individuals from lower social and educational backgrounds significantly undermines the privileges typically associated with their legal status as our analysis intends to show in the following paragraphs.

## 3 Empirical context and methodological framework

**Figure 1:**
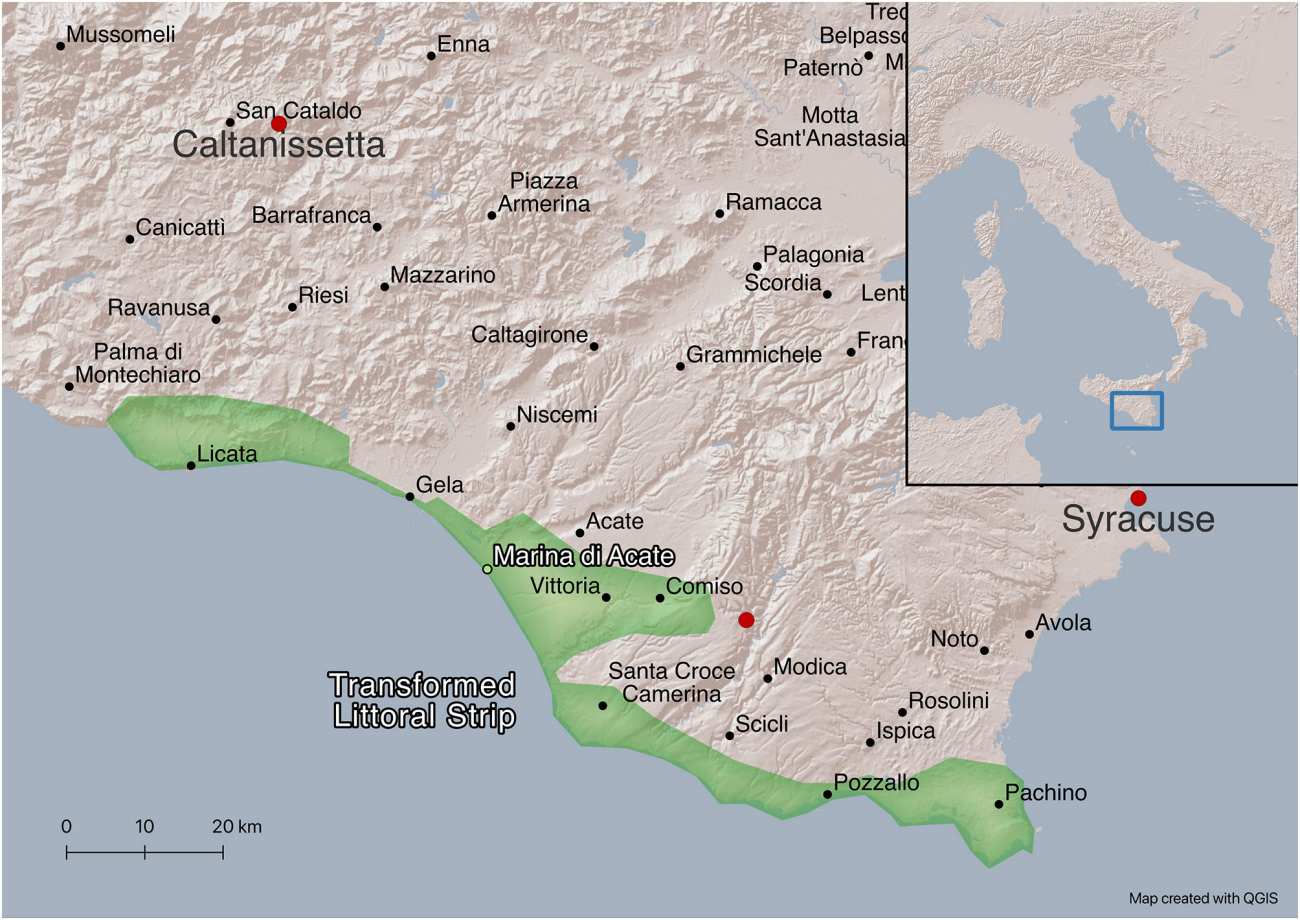
The transformed littoral strip.

Our research was conducted in the part of South-Eastern Sicily known as the Transformed Littoral Strip-TLS—a sandy land that spans multiple villages and skirts the sea for about 150 km—which represents one of the largest greenhouse areas in Italy, known for its vegetable production, especially high-quality tomatoes commercialized all over Europe. The area has undergone profound transformations during the past 70 years. The introduction of greenhouses as of the 1950s has significantly altered the coastline and changed the overall natural ecosystem (Aureliani, 2024) as well as the organization of agricultural production and labor process, now fully de-seasonalized, since intensive agricultural production and harvesting are usually carried out for 11 months per year (Piro, 2021; Cabras and Massari, 2023). In addition to impacting the land and the environment, the transformations occurred in the area have strongly affected social relations as well, due to the dynamics imposed by global food supply chains, with growing international competition, marginalization of local growers, reduction in their profits and sharp lowering of labor costs. The constant need for a cheap workforce has become a structural productive factor, as in other Mediterranean countries (Corrado et al., 2016; Molinero Gerbeau and Avallone, 2016), and has led to an increased informalization of work relations and growing ethnicization of the labor market, where migrants currently account for about 50% of the agricultural workforce of the area (CREA–Consiglio per la Ricerca in Agricoltura e l'Analisi dell'Economia Agraria, 2024, p. 17) (Figure 1).

The TLS, traditionally associated with emigration, since the late 1970s has turned into a destination for migrant workers, initially Tunisian men who gradually substituted internal migrants coming from other parts of Sicily and later, since the enlargement of the European Union in the period 2004–2007, a place of destination for large numbers of Polish and Romanian migrants (Piro and Sanò, 2019; Gentile, 2021). According to Caruso and Corrado (2015), the small village of Marina di Acate, found at the core of the local agro-industrial district—and where our fieldwork activities were mostly carried out—was in the early 2010s the leading city in the Center-South of Italy by percentage of migrant population.

In that decade, however, a significant change occurred not only in the nationalities but also in the gender of the workforce employed in the greenhouses and in the related packaging sector, with an increase in the number of Romanian women hired. Although the actual number of foreign workers employed is hard to estimate,^1^ due to the large amount of informal work, the visibility of Romanian women has started to increase since then, especially in the rural areas near the greenhouses where they are accommodated by the employers, either alone or with their partners, children, or extended families, in some cases reassembled in that complex migration context. Thus, the workplace and domestic environments often overlap, with a consequent lack of autonomy and privacy due to the employers' constant presence and control and a condition of serious spatial and social isolation given the remoteness of these areas from urban centers, the lack of public transport and the obstacles in leaving the countryside without a private car (Piro, 2021; Massari et al., 2024).

The situation of live-in farmworkers has made these women a very hard-to-reach population that is difficult to access due to their spatial isolation, lack of social networks, extremely long working hours, fear of their employer's surveillance and other negative repercussions on both their work and private life. Moreover, their overall social position—cutting across gender, nationality, class and personal status—is affected by vulnerabilization processes which expose them to the risk of suffering further forms of harassment and abuse by their Italian employers, as emphasized by a number of studies that have brought to light cases of sexual blackmailing and abuse affecting Romanian women living in the area (Palumbo and Sciurba, 2015, 2018). Hence, their status of (European) migrant farmworkers employed in a highly exploitative sector is also worsened by both the gendered nature of their exploitation—which is based on prevailing gender norms and stereotyped assumptions over women's bodies and abilities—and by a situational vulnerability which is enhanced by the intertwining of structural and personal factors (Palumbo, 2022, 2024).

Given the empirical context and the sensitivity of the topic, and in line with our ethical approach aimed at minimizing any risk to our project's participants, we began with a preliminary period of field observation. This phase informed our decision to adopt a gradual and reflexive approach to fieldwork, which prioritized the emergence of informal, unstructured interactions as a vital component of the data collection process. Accordingly, we opted for a qualitative method based on both in-depth and semi-structured interviews fully recorded and transcribed (n: 14), and meetings and more informal discussions and conversations (n: 82). Although no interpreters or community researchers were formally involved, access to the field was facilitated through the establishment of relationships with key local actors, which played an important role in shaping the dynamics of data collection and interaction with participants. As far as interviews are concerned, they mostly involved members of local associations and cooperatives, trade unions, cultural mediators, social workers and volunteers engaged in projects and activities carried out in the TSF area aimed at providing support and assistance to migrant farmworkers employed in the greenhouses and their families, a prosecutor involved in investigations related to the exploitation of migrant farmworkers, a local entrepreneur and one migrant woman with a former experience as farmworker in the area. Interviews with Romanian farmworkers-−7 women and 1 man—occurred during more or less casual visits and go-alongs in their domestic and workplace. As regards informal meetings and conversations, they involved a wide array of people, mostly belonging to the same categories listed before, but also including experts, religious leaders, members of local institutions, law enforcement officers, and 11 Romanian women and 2 men. Among the latter 3 women and 1 man were almost silent during our encounters. Moreover, fieldwork activities, which lasted from January 2023 to May 2024, foresaw participant observation and a biographical creative workshop with migrant women (Romanian and Tunisian) based in the area. Overall, we engaged more than 90 participants in our research who, from different perspectives and based on their professional and/or biographical experiences, provided a wide array of information on the main topic of our study, i.e., living and working conditions of migrant women employed in agriculture in the TSF.^2^ The data collected through the interviews and the informal conversations, participant observation and laboratory activities were subjected to a comparative analysis that highlighted some thematic clusters that guided the interpretation phase of the research. In this regard, the division of labor within the research team was shaped by both methodological and analytical complementarities. While some members took the lead in conducting fieldwork—spending extended periods in Vittoria and gradually building trust with research participants—others focused more on theoretical framing. Crucially, methodological reflexivity and data analysis, however, were undertaken collaboratively, through shared moments of reflection and dialogue. These exchanges were vital in maintaining a continuous interplay between the empirical and theoretical dimensions of the project.

## 4 Findings

This part addresses the living and working conditions of Romanian farmworkers in the Sicilian countryside, with a focus on the relationship between citizenship and marginalization. It explores how spatial isolation intersects with various forms of social exclusion—linked to gender, social stratification, and recurring processes of racialization and sexualization—within both work and everyday life contexts. Our analysis aims to bring to light their lived experiences along with the paradoxes and contradictions attached to their formal EU citizenship vis a vis their actual feelings and condition. It also provides an analysis of how these women navigate and challenge oppressive conditions by performing a number of acts and strategies aimed at negotiating benefits to manage exploitation or eventually limiting and/or stopping it. Although these actions are not usually performed in a public way and do not address the underlying structural inequalities, they allow women to retain some control over their lives.

### 4.1 Living and working conditions of Romanian farmworkers in the Sicilian countryside

Multiple forms of vulnerability affect Romanian women employed in the agricultural sector in eastern Sicily. Despite their legal status, Romanian women farmworkers with European passports experience extremely precarious working and living conditions, corresponding to various forms of exploitation and discrimination. The greater or lesser capacity of certain groups to perform citizenship depends in fact on their position along a continuum of exclusion and inclusion, in which holding formal citizenship, as Romanian women do, is not sufficient to obtain and claim such substantive rights. As the research shows, several factors intervene to widen the gap between legal and lived dimensions of citizenship, placing Romanian women farmworkers within social and labor contexts according to hierarchies that only partially reflect their legal status.

Less unionized than other groups of migrant workers, e.g., Tunisian ones, and without the need for a labor contract to legally reside in Italy, Romanian migrants are subject to the same, and sometimes worse, working conditions imposed by local employers on other migrant communities employed in the same agricultural sector. The presence of only formally legal contracts is a tried-and-tested device for lowering labor costs and being competitive in a sector where large-scale distribution limits earning potential, according to the testimonies of local agricultural employers interviewed. These types of contracts, which allow access to agricultural unemployment, enable farmworkers to “recover” the part of their wages not paid by the employer for the hours actually worked (and not contracted) in the form of a state subsidy. In a labor market characterized by a system of highly asymmetrical interactions (Piro, 2021), almost the entire workforce, including Romanian male and female workers, is employed on fixed-term contracts for the minimum number of days to qualify for agricultural unemployment benefits, working, however, as many as 11 months per year (Cabras et al., 2024). According to a local employer:

“It is a gray contract because I give them 180 days, but they actually work about 280 days a year. Then, by contract they are supposed to do six and a half hours a day and they actually work eight [hours a day]. Everyone works eight, but the contract says six and a half. If I pay them 180 days for six hours of work per day [while they actually work eight hours per day]... they manage to get unemployment benefits... because if they exceed that income, they cannot apply [for unemployment].” (Employer, December 10th, 2023).

In addition to the presence of “gray contracts” that are only formally regular, Romanian women employed in greenhouses must cope with a condition of strongly gendered wage inequality, often representing the last link in a production chain whose wages are generally significantly lower than those of the now residual Italian male farmworkers and the Romanian and Tunisian ones.

“The arrival of the Romanian community [...] allows the woman to work in the greenhouse together with men... whereas in the past the environments were quite, let's say... separate [...]. That work, it's a poor job, it's a job that doesn't... and I'm talking to you about the years when women earned 25 euros, it was exactly 50% of the pay... men 28 and Italians 30-33 euros...” (Trade unionist, September 5th, 2023).

Extending the analytical outlook from the purely work dimension of migrant women to living conditions as a whole, the scenario is much more stratified. As already mentioned, employers, referred to by the workers as *padroni*, i.e., “masters,” in addition to employment, usually provide accommodation for their employees. These are often dilapidated dwellings close to the greenhouses, difficult to rent on the local housing market, unhealthy places plagued by widespread air and soil pollution linked to the mismanagement of agricultural and domestic waste:

“The arrival was traumatic, the working and living conditions were difficult. Both of us [she and her husband] worked in greenhouses and lived with another couple in a house that was a kind of garage. One night when I came home to go to sleep, I found a snake and mice in the house. I tried to sleep and not think about it until then, but the conditions were unbearable...” (Former Romanian woman farmworker, March 1st, 2023).

“There is a house that maybe you [the owner] would never have dreamed of renting [given its precarious conditions]; now they can rent it... with mold, with anything... they [migrant farmworkers], in the end, out of desperation, agree to rent it, to have a roof over their heads... but these are houses that have been closed for years and are finally open...” (Social worker, April 28th, 2023).

In a system where the “master” not only provides a formally regular contract, but also accommodation, a domicile where they can legally reside, relations of domination and capillary control over working and living time are on the agenda. Often single and with dependent children, struggling to reconcile production and reproduction work, these women are usually at the mercy of their employer for the management of all daily activities (errands, medical examinations, shopping), given the lack of public transport to the nearest town from their secluded hamlets. Sometimes Italians living in the area are the ones who provide migrants with transport services in exchange for money or rent houses that would otherwise remain empty due to their poor conditions:

“Getting to school is impossible without a car. Initially [her daughter] was accompanied by M., a compatriot. When she was in second grade, however, there was an Italian lady who accompanied S. and three other children in exchange for 40 euros per week. At a certain point, the other children no longer needed the ride, and the lady was no longer available.” (Romanian woman farmworker, December 2nd, 2023).

This is therefore a condition of spatial and social isolation that manifests itself in ways that appear functional to the reproduction of a *differential inclusion* (Mezzadra and Neilson, 2012), i.e., it appears useful to maintain certain power relations and to consolidate an idea of citizenship, conceived here as a social process, which in the Marshallian sense does not protect against racializing and extractivist practices underlying a general and multidimensional system of exploitation.

As the fieldwork shows, the case of Romanian women is particularly paradigmatic in this sense. The availability of European citizenship—this bureaucratic “privilege” discussed above—does not constitute an instrument of protection. Instead, it makes these women “available” to adapt to working and living conditions even more unbearable than those imposed on other migrant groups (e.g., Tunisians).

The presence of situated and stratified forms of vulnerability thus weakens the ability to guarantee rights on the basis of citizenship. This results in a scenario where a segment of the population—Romanian women—is formally integrated from a legal-administrative perspective, yet faces discrimination in both work and everyday life. They remain socially and physically invisible and are exposed to various forms of gender-based violence and sexual exploitation. Thus, Romanian women experience a lessened citizenship (Karagiannopoulou et al., 2024), which however does not exclude acts of resistance from below in the field of everyday life.

### 4.2 Gender discrimination and body exploitation

Most of the research on the exploitation of migrants in agriculture has been affected, especially in the past, by a sort of gender neutrality, whereas farmworkers' experiences, practices, identities and roles, as well as demands, struggles and counterstrategies have been often addressed without paying attention to the distinctive mechanisms shaping and governing men and women's work and life (Palumbo and Sciurba, 2018; Sexsmith and Griffin, 2020; Palumbo, 2024). This has caused a significant invisibilization and/or oversimplification of the different impact created by a number of features characterizing contemporary forms of racial capitalism (Robinson, 1983)—such as wage inequalities, irregular work contracts, exhausting working hours, forms of harassment and abuse, multiple exploitation, discrimination and racialization practices—on male and female migrant farmworkers. As outlined before, our case-study confirms that gender and nationality function as a mechanism of separation and social stratification which is strictly connected with the needs of production. Romanian women employed in the greenhouses in the TLS and living in secluded areas once associated with the historical backwardness of Southern Italy are de facto embedded in gendered and racialized hierarchies which are not alien to the dynamics of capitalism, but central to its functioning and further expansion.

Furthermore, engendering migration and exploitation leads us to turn our attention to the different ways in which male and female farmworkers struggle among the various constraints affecting their daily life, as well as to the attempts to survive a highly oppressive context and/or resist a number of obligations which may dramatically impact their migration process and its related expectations. Both in the origin and destination countries, gender relations and well-rooted and interconnected patriarchal structures of power produce multiple forms of oppression which affect women's choices and forms of mobility along precise “gendered geographies of power” (Pessar and Mahler, 2003). The decision to migrate is often made within a wider context of gendered interactions and expectations where women suffer forms of social, economic and political marginalization in their country that still persist in the hegemonic system of gender stratification they find in Italy.

In a purely male-dominated agricultural sector—the employers are in most cases Italian men—organized around spatially isolated greenhouses, Romanian women farmworkers are often subjected to severe forms of exploitation that include incessant working hours, in the absence of safety devices. Within a confined, invisible, and constrained workspace, i.e., greenhouses, they are forced to work in the cold during the winter months and in the exhausting heat of the summer, sometimes with serious health consequences:

“After being fired yet again as a caregiver [...] I found myself working in a very isolated greenhouse. I just worked… At that time, I didn't have a phone or a watch... I didn't even know how many hours I was working in the greenhouse.” (Romanian woman farmworker, December 2nd, 2023).

“The most frequent diseases are osteo-articular ones... linked to the workload, to the sometimes incongruous positions that cultivation requires... and then clearly to the temperature changes, there is heat, humidity, cold in the greenhouse... in summer it reaches and exceeds 40 degrees [...] or pathologies in the respiratory allergic sphere [...] both for products, for phyto-pharmaceuticals and also for seasonal allergies.” (Physician, April 10th, 2023).

Women are also sometimes considered better suited to perform certain precision tasks: their hands are prized in meticulous work according to a stereotypical definition of female labor in which the (supposed) propensity for care and precision compensates for the (supposed) lack of strength and physical endurance:

“Even if they are women […] in some production phases they even work better, because for planting a woman is better than a man, because their hands are more tapered, precise […]. The local masters, steeped in patriarchy, find themselves in the ideal condition of having goods, blackmailable and exploitable flesh within their own company, away from the hassles of their wives. This is the situation, it's the perfect context for committing a series of crimes and abuses against a person.” (Social worker, April 27th, 2023).

Wearisome work in greenhouses, whose tasks often require debilitating postures and physical exertion during cultivation and harvesting, is accompanied by various forms of violence that include sexual harassment and constant blackmail by employers:

“One girl related that as soon as they [the employers] go to work they immediately ask for ‘something else' [sexual services]. She had never had a contract and always left to escape this situation. Sometimes these things happen with the husband's consent, in order to have the contract. Or these things also happen with the employer's friends.” (Romanian nun, February 2nd, 2023).

The forms of exploitation experienced by Romanian women farmworkers are highly differentiated and deeply gendered, shaped by their specific social and economic conditions. In addition to receiving lower wages, these women are exposed to repeated practices of racialization and sexualization, which render their bodies available to employers and reinforce multifaceted relationships of dependence. Indeed, in the case of Romanian women, sexualization acts as an instrument of discipline “when applied through racial distinctions” (Krivonos and Diatlova, 2020, p. 127). The racialized femininity of Eastern Europe serves to reaffirm the norm of Western and Northern white femininity, according to well established dichotomies. As was pointed out above, the femininity of Romanian women is seen as “other,” dangerously provocative and therefore less respectable than that of Italian women. As the research shows, the association “Romanian woman-prostitute” is very frequent in the common imagination:

“They treat you as their property. You are property. He [the employer] was hitting on me at first not in front of my husband, then even when my husband was there. Then I decided to leave because I couldn't take it anymore. I couldn't stay like this. They don't think you are a worker; you are a property. Then he also controlled my movements, he asked me why I was going to the village. The people who know me here, even the ladies, know what I am like… They think all Romanian women are whores.” (Romanian woman farmworker, December 2nd, 2023).

That perverse mix of sexism, racism and patriarchalism that often informs employers' behaviors and practices is further evidenced and amplified—as the interview mentioned before highlights—by the recurrence of forms of humiliation even toward foreign men, sometimes obliged to passively assist to their partners' harassment.

### 4.3 Agency practices and forms of survival: reflexive flexibility, escape and rupture

Despite the intersecting axes of oppression—rooted in gender, class, ethnicity and family/personal status—that shape the lives of Romanian women farmworkers in eastern Sicily, our research sheds light on the counteractions that these women display mostly in their everyday life and their agency practices, even in their precarious situations (Lutz, 2015; Basok and Bélanger, 2016; Schenk, 2020). Their embodied experiences of marginalization give rise to situated survival strategies that are not merely reactive but also seem to be shaped by women's reflexive awareness of their positionalities within overlapping systems of power.

Analyzing the complex interplay between situated vulnerability and the strategies adopted to mitigate related risks can shed light on the range of choices available to migrant women, especially in contexts where formal rights are granted by state policy and legislation but denied in practice. Adopting a perspective that avoids both victimizing narratives and overly romanticized or heroic accounts of migrant women's agency can help illuminate how the Romanian women in our study construct their own life trajectories, primarily through micro-practices embedded in their daily work and lived experiences. Keeping in mind a basic definition of agency as the ability to exercise some degree of control over the social relations one is involved in, which in turn also implies the ability to *transform* those social relations to some degree (Sewell, 1992, p. 20), our research, also building upon previous studies carried out in other work sectors (Rydzik and Anitha, 2020), showed the emergence of various strategies designed to challenge and/or transform subaltern social relations through continuous processes of renegotiating disputed meanings and often denied rights.

Drawing on the experiences of our research participants, as well as interviews carried out with volunteers and social workers, agency emerges primarily as an act of *reflexive flexibility*. It is expressed through the capacity to navigate and endure oppressive living conditions and exploitative labor arrangements, contexts that are often resistant to structural change. This may take the form of apparently discreet and passive strategies, such as in the case of those women who continue to work, even under unbearable conditions, and keep quiet but then turn to alcohol as a form of temporary escape or relief from the emotional challenges and anxiety associated with their situation. This form of self-help, however, adopted to cope with work and life's pressures and emotional pain, may also lead to a problematic use that affects their daily life—especially the relationship with their children and, in general, their maternal role—and wellbeing, thus aggravating their already fragile condition (Romanian nun, February 2nd, 2023). Alcoholism is a problem that affects both female and male farmworkers based in the area, as a health operator underlines:

“(…) alcoholism, which for some can be considered a sort of self-treatment. Because at a psychological level there are symptoms of anxiety and depression linked to the limbo situation they have experienced and are experiencing, the lack of documents. Some say, ‘after ten years it's as if we arrived yesterday.' They are anxious because they think about future and they do not see it.” (Health operator, 13th March, 2023).

This capacity to expressly use individual *flexibility* as a way of survival and conscious control over oneself, avoiding total subjugation, can also manifest through more subtle tactics. These are often aimed at defending themselves, at least, from the most evident sources of vulnerability associated with being perceived as a migrant, subaltern, isolated, vulnerable woman, i.e., the risk of being sexually harassed, as previously discussed. As a nun based in the area and involved in social projects expressly addressed to migrant women and children living in the small village of Marina di Acate emphasizes during a conversation with us, in places like these “it is difficult to be alone” for a single migrant woman, because of the ongoing risk of being sexually assaulted by her employers (Romanian nun, February 2nd, 2023). This is the reason why, according to her, some Romanian women tend to establish a relationship with male partners—whether they are Italian, co-nationals or foreigners—as a form of apparent utilitarian “protection,” even though a very ephemeral one. The fragility of this kind of solutions also emerged during a conversation with a Romanian cultural mediator, a former farmworker, who emphasizes how even married women are not *protected* from this risk:

“The Italian employer arrives and tells your partner ‘you stand aside, I have to have a discussion with your wife.' All this, for 40 euros? Not sex, often a grope. They are old, for them it's enough just to look at you. But if you do it once, it's over…” (Cultural mediator, March 1st, 2023).

Tactics aimed at coping with the widespread request to provide sexual favors to employers may also imply the attempt to negotiate some benefits related to their apparent submissiveness. This finding also emerged during the same conversation with our research participant, now a cultural mediator, who recalled that in return for the recurrent gropes from her “master,” she used to claim some cigarettes packs that carved out, at least, a sort of control over her body. Far from normalizing sexual abuse, this coping tactic helped to distance herself from the emotional pressure associated with these encounters, to instrumentally play the role of the docile subject just to achieve non-work-related benefits and to carry out a day-to-day survival in the face of the oppressive and stressful conditions she was experiencing at that time.

As in the study carried out by Rydzik and Anitha (2020) among Eastern European migrant women employed in the tourism sector in the UK, similar tactics have often been referred to in terms of resilience. This is, however, a largely abused term in the analysis of social phenomena and in common language, while it is also framed into neoliberal rationality, thus risking conveying a normative regressive idea of acceptance of structural inequalities and power relations (Diprose, 2015). What both reflective *flexibility* and *resilience* display, nevertheless, is an emphasis on an overarching individualized logic driving women facing a very diverse array of challenges and adversities in their workplace. These acts, while not eroding the inner, structural and pervasive factors that shape and affect their hard conditions, at least allow their day-to-day survival and struggle to desperately maintain an even weak sense of control over their life.

Besides being reflected in actions aimed at mitigating abusive and exploitative conditions on a short-term basis, other forms of agency may attempt to directly recognize and address these conditions and find alternative solutions in the medium or long term, at least at individual level. Previous studies carried out in other areas and work sectors have already shown that migrant workers often opt for “pragmatic ways to evade and reconfigure one's location within the prevailing hierarchies without seeking to dismantle them” (Rydzik and Anitha, 2020, p. 891). One of these ways is quitting a job, seen as a form of subtle resistance aimed at escaping exploitative environments.

In the empirical context explored here, quitting a job in a greenhouse may represent an *escape act* as result of the survival strategies carried out by exploited women in the countryside. As reported by Alberti and Sacchetto (2024, p. 38), contrary to the common view in organizational psychology that resignation represents an emotional and immature response of workers to unsatisfactory conditions (March and Simon, 1958), other authors (Edwards and Scullion, 1982) have explicitly recognized the possibility that quitting can represent an expression of agency and conflict. However, in the case of Romanian women, this does not entail a collective dimension. As a young Romanian woman worker points out, recalling the sexual favors demanded by her employers:

“You know, at the beginning everything was fine, then the bosses see you are a foreigner, and they don't care that they are married, that they have families, and they always try. I changed many jobs because of this, until I found a good one.” (Romanian woman farmworker, December 2nd, 2023).

Quitting thus allows these women to regain a space of autonomy and apparently to free themselves from widespread forms of exploitation, which often become intolerable. In a context of living and working isolation, which generates immobility, the possibility of changing employment—either in the same area or elsewhere—can also offer the opportunity to overcome the condition of marginalization. Finally, these practices openly question the *masters*' assumptions that migrant women are always available and willing to work in unfavorable conditions.

As emerges from the fieldwork, some Romanian women experienced other employment both before and after their entry into the agricultural sector, starting with caregiving. The care sector is in turn an area particularly exposed to forms of exploitation and subalternity for the women employed, as one research participant who had been living in Italy for some time emphasizes:

“When I arrived in Sicily, in the early years I worked as a caregiver in farming families. The conditions were difficult because the work was short, and I was fired without notice. Italian employers threw me out of the house without notice. After I was fired yet again as a caregiver, I met other Romanians who told me about the greenhouses…” (Romanian woman farmworker, December 2nd, 2023).

Another alternative to agricultural work concerns the sex work market. Informal conversations during our research reveal that some women manage to combine agricultural work with sex work. In other cases, however, labor conditions can become so exhausting that sex work is preferred to working in greenhouses. Quitting agricultural labor may therefore become a form of *escape* from a specific set of power relations and a sort of *reworking strategy* (Scott, 1985; Rydzik and Anitha, 2020). While these practices may challenge employers' assumptions about migrant women's willingness to work in poor conditions and suffer sexual harassment, some stories collected during our fieldwork highlight sex work as an option to escape poor conditions. By escaping hyper-exploitative and severely degraded working conditions in the greenhouses, however, these women reassign and reallocate themselves to very similar working conditions and power dynamics.

Moreover, in the case of farmworkers with European citizenship, these forms of “hypermobility” (Bolokan, 2023)—where workers frequently change their labor arrangements, moving between different farms, factories, or even job sectors—often imply regular returns to their place of origin. In this regard, it might be emphasized that caregiving duties—both transnational and local—significantly shape women's availability for work, their mobility patterns, and their negotiation of precarious employment. These responsibilities often constrain their choices, reinforcing gendered labor segmentation and limiting access to more stable or better-paid opportunities. In our research, this was particularly evident in the case of Romanian women living with their children. Thus, hypermobility is not just a mere response to labor market demands but is also deeply embedded in the intersection of gendered expectations and familial obligations (Palumbo and Sciurba, 2018). Moreover, as already emphasized by Perrotta (2015) with reference to Romanian farmworkers employed in Southern Italy and still confirmed by the outcomes of our research in Sicily years later, Romanian workers tend to accept lower wages and poor labor relations because their *ability to escape*, i.e., going back and forth from Romania or moving to other countries, is probably their ultimate and most powerful form of survival and a true act of agency. Hence, especially in the case of those women who commute on a regular basis between the two countries, spending half year in one place and the remainder in the other, physical and social immobility *and* hypermobility can eventually coexist (Bolokan, 2023). Thus, although legal female farmworkers coming from European semi-peripheral regions are often in very precarious conditions, their life and wellbeing are largely affected by their differential inclusion into the work market due to the intertwined effect played by widespread racialization and sexualization processes.

Finally, a third set of responses may go well beyond the “everyday forms of resistance,” to quote Scott's (1985) well-known expression outlined above, and position themselves as actual acts of *rupture* aimed at claiming rights and, at same time, engaging in practices of citizenship that may even lead to challenge and bring about changes in employment relations and related power structures. Thanks to the crucial support provided by grassroots organizations and trade unions, one of the Romanian women we met in Sicily during our fieldwork—currently a cultural mediator working for a local NGO—managed to break out of the situation of profound exploitation and isolation she and her family had being living for years. As she tells us: “I left, I found the courage. I left and found the courage to press charges against my employer” (Cultural mediator, March 1st, 2023). By reporting her employer, our research participant not only dared to challenge her *master*'s power, but also established herself as an individual with the rights and capacity to change a situation that can have a collective impact for other farmworkers as well. Although this was the only act of rupture that we had the chance to evidence during our fieldwork, the story that our research participant told us emphasizes how these acts are not necessarily spontaneous, but might be crucially facilitated by associations and groups engaged at grassroot level that provide not only material assistance but also spaces for recognition, collective voice, and legal empowerment. By tracing these pathways, this analysis highlights how agency is relational and contingent, shaped by solidarities that fortunately challenge the exclusion from the European privilege and reconfigure the boundaries of lived citizenship.

## 5 Discussion and concluding remarks: living and performing citizenship at the margins of European whiteness

The idea of citizenship is often, more or less consciously, associated with the city, considered the place where even the most marginalized subjectivities can find spaces for common action and claim rights and recognition. Many analyses of citizenship practices and acts, therefore, tend to overlook the experiences of those entangled in social marginality combined with spatial isolation, which fosters forms of subjugation and domination. In this article, we have explored how subjectivities that are formally recognized as part of a common European citizenship, but navigate conditions of extreme poverty and socio-spatial isolation, experience their citizenship in the rural context of Southern Italy. We have examined the forms of inequality around which their subalternity is intertwined, as well as the nuanced forms of agency they enact, even within a context of social and political invisibility. The lives of the Romanian women farmworkers we encountered in the TLS, either personally or through the attentive accounts of the few social actors living in the area and interacting with them, highlight the existence of lived forms of citizenship despite only partial access to the rights they should be entitled to, as the literature on lived citizenship has extensively emphasized. However, they also reveal how, conversely, forms of transnational citizenship that guarantee rights even in the absence of national citizenship, such as European citizenship, can counterintuitively become entangled in power dynamics and intersect with forms of racialization and sexualization at the root of discrimination and domination.

Citizenship functions as an ambivalent device, whose conceptual matrix must be contextualized within the framework of European colonial history and the strongly Eurocentric vision that supported it. Indeed, citizenship necessarily introduces a divisive logic between those who are included and those who are excluded from it. However, within a system of racial capitalism—which functions through the construction of differences between groups, and even among individuals within the same group, as happens within different grades of whiteness (Kalmar, 2023)—the possession of citizenship, under certain conditions, can actually facilitate exploitation. Specifically, European citizenship, when combined with East-West migration dynamics, becomes a key mechanism through which certain forms of exploitation are legitimized and sustained. In the case of Romanian women farmworkers, European citizenship perfectly responds to certain needs of capitalist production, so much so that the “bureaucratic privilege” enjoyed by Romanian migrants is transformed into their willingness to accept particularly onerous working conditions and little inclination to claim certain rights, as our analysis has shown. On the one hand, irregularly recruiting EU citizens into the labor market poses a lower risk for employers, as they are not subject to criminal penalties, unlike the employment of non-EU migrant workers. On the other hand, this bureaucratic privilege is significantly weakened by the lack of a strong and organized community—such as the Tunisian one, which is often more unionized—and by gender-related vulnerabilities. These factors expose them to the intersection of labor exploitation and gendered discrimination and violence, further limiting their possibility to actually enjoy their citizens status and pushing them to accept harsh living and working conditions.

Even though European citizenship guarantees freedom of movement—the possibility of crossing borders, living regularly within the EU, and migrating from one EU state to another based on personal choices and constraints—Romanian women farmworkers, regularly resident in Italy, experience a persistent spatial, social and symbolic immobility. This confirms that the condition of immobility, in its various forms, is part of the migratory experience, which is inherently mobile (Bélanger and Silvey, 2020), despite the formal possibility of movement within the territory of residence and across national borders. In this context, spatial isolation and the conflation of domestic and work spaces—as emerged in our empirical findings—contribute to minimizing the structures of opportunity for Romanian women and pave the way for labor exploitation and sexual abuse. These are not merely juxtaposed but, being intertwined with racializing processes, become conditions for each other.

However, domination and oppression are only one aspect of the situation. From our research, despite the structural limitations of accessing the voices of a population that is extremely intimidated and made dependent on the power apparatus we illustrated, it clearly emerged that Romanian women farmworkers in Sicily operate identity and material repositioning, reaction, and withdrawal, which the lens of lived citizenship allows us to grasp in its political dimension.

Our research shows that discrimination and marginalization do not revolve solely around the citizen-foreigner dichotomy. As citizenship is increasingly lived transnationally, these dynamics are shaped by a range of intersecting factors—including gender, sexualization, and racialization—that extend beyond the Manichean division between white and black. Notably, these processes also operate subtly within the signifier of whiteness itself. Romanian working-class women in Northern and Western Europe are positioned at the margins of whiteness, with their place of origin hierarchically situated at a lower level on the European scale, due to common racializing dichotomies of civilization/rationality vs. barbarism/irrationality. In our research, this becomes even more meaningful, as it occurs in a location—Southern Italy—that has experienced similar processes of internal othering within the Italian context (Conelli, 2022).

All these nuances contribute both to persistent forms of exploitation and subjugation, paradoxically linked to the status of European citizenship, and to new ways of feeling oneself as a citizen or performing citizenship. In this sense, as our research shows, obtaining small benefits or leaving the workplace, fleeing from the farms, can be ways to assert one's agency, these are tactics that subtly subvert regulations or challenge the various forms of discipline employed to control the bodies of migrant women workers (Candiz et al., 2023). In this regard, the hypermobility exhibited by Romanian farmworkers within the migratory space connecting rural areas in Southern Italy and Romania can be understood as a reaction to subjugation, immobilization dynamics, and spatial isolation. This form of mobility can be seen as a practice of transnational lived citizenship, where spatial, intersubjective, performative, and affective dimensions intertwine (Kallio et al., 2020).

Finally, for Romanian women, it is therefore possible to seek better opportunities, by circulating in the three main work areas accessible to them, namely agriculture, care, and sex work. In particular, agriculture and sex work are configured both as complementary options and as alternatives, where sex work is often seen as the lesser evil. These forms of action are not organized or particularly effective in terms of individual and collective emancipation and are performed within a very stringent system of constraints.

In this regard, analyzing migrant women's agency within this highly exploitative sector allowed us to deconstruct that sort of gender neutrality that affects part of the research on the exploitation of migrants in agriculture and to critically reconsider a linear and univocal view of migration as necessarily emancipatory (Schmoll, 2024, p. 174). Nonetheless, their situated vulnerability does not correspond to passivity and inability to make decisions about their life, while it can become a generative device of crucial decisions and acts. Rather than powerless victims of racialized and sexualized exploitation and work regimes, these women—who are European citizens—display nuanced and interconnected forms of survival and resistance that deserve greater attention, as they struggle to assert *their* rights and agency within a citizenship framework that still remains ambivalent and contested.

## Data Availability

The datasets presented in this article are not readily available for confidentiality reasons. Due to the strict confidentiality offered to research participants, no datasets of interviews will be shared without extra consent by them. Requests to access the datasets should be directed to monica.massari@unimi.it.

## Footnotes

^1^According to the latest official data available, in 2023 Romanian migrants accounted for around 23% of the overall migrant population in Sicily. In the area covered by our research—i.e., the province of Ragusa—Tunisia represents the first country of origin (around 9,800 migrants mostly employed in agriculture), followed by Romania (around 7,100 migrants): see https://www.tuttitalia.it/sicilia/provincia-di-ragusa/statistiche/cittadini-stranieri-2024/.

^2^Our research is part of project PRIN2020-MOBS—*Mobilities, solidarities and imaginaries across the borders: the mountain, the sea, the urban and the rural as spaces of transit and encounters* funded by the Italian Ministry of University and Research. For more information on the project and its activities see: https://mobsprin2020.org/. The protocols and research activities implemented by the University of Milan's team were approved by UNIMI Ethics Committee on 18 January 2023 (approval no. 9/23). Moreover, the theoretical framework of our analysis highly benefitted from discussions carried out by the research group in the framework of Horizon-MSCA-2021 MEMODIAS—*Memory Practices of the Afghan and Somali Diasporas in the USA and Italy*.
